# Sense of Coherence in the Perinatal Period: A Longitudinal Growth Mixture Modeling Analysis

**DOI:** 10.1002/jclp.70156

**Published:** 2026-06-06

**Authors:** Kelsey Perrykkad, Stuart Watson, Andrew J. Lewis, Marinus Van IJzendoorn, Megan Galbally

**Affiliations:** ^1^ Psychiatry Department, The Centre for Women's and Children's Mental Health Monash University Melbourne Australia; ^2^ Monash Centre for Consciousness and Contemplative Studies (M3CS) Monash University Melbourne Australia; ^3^ Health Futures Institute Murdoch University Murdoch Australia; ^4^ King Edward Memorial Hospital Subiaco Australia; ^5^ Institute of Health and Wellbeing Federation University Ballarat Australia; ^6^ Mental Health Program Monash Health Melbourne Australia

**Keywords:** individual differences, major depression, maternal childhood trauma, matrescence, perinatal mental health, sense of coherence

## Abstract

**Objectives:**

Previous studies have established that higher *Sense of Coherence* (SoC) predicts lower pregnancy‐specific distress, fewer delivery complications, and increased birth satisfaction. However, less is known about how SoC typically changes over pregnancy, birth, and postnatally and the risk factors and protective factors contributing to SoC trajectory during the perinatal period. In this study, we aim to describe and predict common trajectories of SoC in the perinatal period.

**Methods:**

680 women in the Mercy Pregnancy and Emotional Wellbeing Study completed measures of SoC on four occasions across pregnancy and the postpartum. Predictors included history of childhood trauma, expectations and outcomes from birth and the postpartum, and postpartum social support. Depression was assessed by structured clinical interview in early pregnancy. Growth mixture modeling was used to classify participants into trajectories of SoC, and multinomial logistic regression models predicted class membership.

**Results:**

Four trajectories were identified: (1) High (67%), (2) Low (21%), (3) Rising (8%), (4) Falling (3%). The strongest predictors of class were depression diagnosis, associated with Low and Rising trajectories, and a history of childhood trauma, associated with Low and Falling trajectories.

**Conclusion:**

Childhood trauma was thus predictive of a poor SoC at 12 months postpartum, suggesting that pregnancy and birth have a negative impact on women's resilience resources, whereas depression in early pregnancy is more predictive of poorer starting point of SoC. Targeted mental health support in the perinatal period may ensure individuals have adequate coping resources.

## 1 Introduction

As distinct from an approach focused on the causal factors resulting in mental illness, Antonovsky developed a salutogenic approach to mental health which aims to discover factors which support the development and maintenance of good mental health and well‐being (Antonovsky 1979). Sense of Coherence (SoC) is the key construct introduced by Antonovsky to describe an individual's orientation to life, which he saw as essential for explaining salutogenesis. Someone with a high SoC believes that the world is worth engaging in, that life proceeds in a mostly predictable way which is under their control, and they are able to make use of available resources to react to life's stressors (Antonovsky 1979). Differences in SoC are thought to explain why stressors lead to poor mental health in some people and are adequately managed by others. SoC has now been shown to predict increased quality of life (Eriksson and Lindström 2007), better mental and physical health in both adolescents and adults (Eriksson and Lindström 2006; Matić and Vuletić 2023), health seeking behavior (Binkowska‐Bury et al. 2016), and the response to personal challenges including health conditions and traumatic stressors (Richardson and Ratner 2005; van der Hal‐van Raalte et al. 2008) in line with Antonovsky's original theory.

In his original descriptions, Antonovsky (1979) identifies SoC as a long‐lasting trait, with only minor fluctuations around a personal setpoint determined by early adulthood, which itself can be altered only by a “radical change in one's structural situation” (p. 125). Despite this, targeted salutogenic interventions have been shown to significantly increase SoC (Langeland et al. 2022), demonstrating that SoC may be a modifiable protective factor, including during the perinatal period (Muggleton et al. 2021). There is mixed evidence about the fluctuation of SoC across the lifespan, with some studies showing stability across ages (Grevenstein and Bluemke 2017; Matić and Vuletić 2023; Richardson et al. 2007), while others show a significant increase in SoC with age (Ferguson et al. 2015; Trap et al. 2016).

Pregnancy and the transition to parenthood is a significant developmental transition and has been described as a canonical personally and epistemically transformative experience (Paul 2014); an apt candidate for a radical change in one's structural situation. Previous studies undertaken in the perinatal period have shown higher SoC to predict lower pregnancy‐specific distress (Staneva et al. 2016), fewer delivery complications (Oz et al. 2009), increased breastfeeding duration (Cortelo et al. 2018), higher rates of vaginal birth and increased birth satisfaction (Ferguson et al. 2016). A recent systematic review of SoC in pregnancy found 49 studies from 14 countries (Alcantara et al. (2023); note preprint status). Given that SoC is predictive of important outcomes and is likely to fluctuate during this transitional period, it is important to consider any common trajectories in SoC in order to better support positive outcomes for families.

Most previous studies measure SoC at a single timepoint, a few compare two timepoints, and there are four three‐timepoint studies. Studies that do measure SoC longitudinally in the perinatal period have shown increases between pregnancy and the early postpartum (< 3 months (mths)) (Bäckström et al. 2018; Ferguson et al. 2016; Hildingsson 2017). Comparisons of postpartum timepoints show mixed results, with one study showing a decrease between 3 and 12 mths postpartum (Hildingsson 2017), while another showed stable SoC between 1 week (wk) and 6 mths postpartum (Bäckström et al. 2018). In notable contrast, Sjöström et al. (2004) and Ngai and Ngu (2016) showed no significant changes in SoC across three timepoints each in the perinatal period. No previous study has considered SoC at four timepoints in the perinatal period (Alcantara et al. 2023) spanning changes within subjects in both pregnancy and the postpartum, thus limiting our understanding of longitudinal trajectories during this transformative period. There have been no complex statistical models of SoC trajectory in this period and no investigation of whether most individuals follow the same trajectory. Analyzing more timepoints gives us the ability to reliably detect and model non‐linear changes to SoC in the perinatal period. Further, a *person‐centered* approach, such as the one taken here, can be used to test theoretically driven hypotheses about common factors shared by individuals who share trajectory class membership, in contrast to traditional *variable‐centered* models which assume variation in an individual's scores reflect variation within one generalizable and normative pattern (Lewis et al. 2020). Our approach allows for better predictive accuracy with regard to outcomes for individuals following similar trajectories.

Factors that should influence an individual's trajectory of SoC through the perinatal period are those which reflect (or buffer against) the radical change in structural circumstances, and how they are perceived by the individual. Since the most dramatic change in lifestyle comes with one's first child (and the initial adoption of the role of “parent”), parity is likely to affect related changes to SoC. Consistent with this, previous literature shows that multiparity is associated with decreased SoC (Przestrzelska et al. 2018; Sjöström et al. 2004). In the original theory, the important elements that would affect SoC are (1) those that affect the perceived comprehensibility of the world, (2) the resources available to the individual to help manage the demands of the situation, and (3) whether the situation feels worthy of engagement and effort.

The perinatal period is typically filled with hopes and dreams for the future (or at least expectations about what the new phase of life will bring), as well as a range of new and unpredictable challenges. Predictability of the environment in early developmental phases when SoC setpoints are being determined, as well as the perceived predictability of changing circumstances during transformative experiences, are both likely to affect the comprehensibility component of SoC. To capture the influence of early experiences on expectations of unpredictability and an individual's sense of control as in the predictive adaptive response model (Troisi 2020), we include a history of childhood trauma as a potential predictor of poor sense of coherence in the perinatal period (Ross and Hill 2002). In terms of experiences in the present, we measure whether expectations about what will happen during birth and in the early months of parenting are fulfilled. As such, reflecting predictability of changes during the perinatal period, we anticipate that more mismatches in expectations and outcomes will lead to falling SoC by reducing comprehensibility.

According to Antonovsky's original theory, one of the determinants of successful tension management is the availability, recognition, and use of generalized resistance resources, which constitute an arsenal of coping strategies which are rational, flexible, and farsighted (Antonovsky 1979). These generalized resistance resources include socioeconomic resources such as wealth, shelter, and food, as well as social support, which is also recognized as a key factor for mental health in the perinatal period (Bedaso et al. 2021; Sufredini et al. 2022). Perceived social support is thus expected to improve SoC or maintain it at a high level in the postpartum. This would also be consistent with previous literature showing a moderate positive correlation between social support and SoC in the perinatal period (Carlsson et al. 2015; Hildingsson 2017; Staneva et al. 2016).

The final component of SoC which may affect its trajectory in the perinatal period is whether life is perceived as meaningful and worthy of engagement. As such, one major factor to better understand low and falling trajectories of SoC in the perinatal period is the presence of perinatal depression, which is characterized by a lack of interest and engagement in daily activities among other symptoms (American Psychiatric Association 2022). Previous literature consistently shows a negative association between self‐reported depressive symptoms and SoC in the perinatal period, using the Edinburgh Postnatal Depression Scale (EPDS) (Alcantara et al. 2023; Ferguson et al. 2016, 2015; Hildingsson 2017; Iwanowicz‐Palus et al. 2021; Przestrzelska et al. 2018), the Beck Depression Inventory (Engelhard et al. 2003; Hittner and Swickert 2010), and the Hospital Anxiety and Depression Scale (Sjöström et al. 2004). The meaningfulness subscale of the SoC‐29 has also been shown to predict scores on the Beck Depression Inventory in depressed individuals (Carstens and Spangenberg 1997).

However, despite this plethora of evidence for an association, no previous study has considered a previous history of depression or included a diagnostic measure of depression when considering the relationship between depression, depressive symptoms, and SoC in the perinatal period. Given that a lack of interest to engage with the world and enjoyment in daily activities is a symptom that distinguishes depression from other closely related affective disorders such as anxiety, the specificity of these screening measures (which often pick up general distress, see Galbally et al. (2023), Perrykkad et al. (2025), and Tendais et al. (2014)) to solidify the conceptual link is lacking. Further, accounting for a past history of depression (and early antenatal depression) rather than postpartum onset of depression is important to account for depression's influence on the trajectory of SoC across the entire perinatal period (conception to 1‐year postpartum). As such, to account for the impact of depression on SoC, we rely on results from an antenatal diagnostic clinical interview.

This study will investigate whether patterns of change in SoC are consistent across individuals, by performing growth mixture modeling to identify latent classes of SoC trajectory between early pregnancy and 1 year postpartum. Our main research questions are (1) are there distinct classes of SoC trajectory in the perinatal period and (2) if yes, can we predict trajectory class membership from enviro‐psycho‐social factors up to and including 6mths postpartum? We hypothesize that the majority of participants will be classified as having stable trajectories of SoC across the perinatal period, consistent with the original conceptualization of SoC as a mostly stable trait. We expect some people to having increasing or decreasing SoC over this period to reflect a change in setpoint during the transformative experience. We also expect group membership will be predicted by demographic factors such as age and parity, matched/mismatched expectations about birth and early parenting, antenatal depression, and postpartum social support.

## 2 Materials and Methods

### 2.1 Participants

The sample in this study is drawn from the Mercy Pregnancy and Emotional Wellbeing Study (Galbally et al. 2017) including 887 participants recruited in Melbourne, Australia, as well as in Perth and regional and rural Western Australia. The study was approved by the Human Research Ethics Committees of Mercy Health (R08/22), the South Metropolitan Health Service (REG Number: 2016‐192), and the Western Australia Country Health Service (18/02) and was conducted in accordance with the approved protocols and the Australian National Health and Medical Research Council's National Statement on Ethical Conduct in Human Research. All participants provided written informed consent.

Women were recruited at less than 20 weeks of pregnancy. Data were collected between 2012 and 2020. This study uses data from four timepoints of this longitudinal cohort, which were collected in early pregnancy (12–20 wks; Wave 1), mid‐pregnancy (~28 wks; Wave 2), 6mths postpartum (Wave 4), and 12 mths postpartum (Wave 5). Wave 3 captures birth outcomes from hospital records. All timepoints containing a SoC measure during pregnancy and the first 2 years postpartum were selected from the broader cohort study to maximize the coverage and detail within the perinatal period for reliable trajectory modeling. These timepoints also give us an equal number of datapoints from before and after birth, so as not to bias trajectory specification and membership towards either pregnancy or postpartum but rather the entire perinatal period.

Participants were excluded from all analyses if they did not have data for at least one timepoint in pregnancy and one in the postpartum, resulting in a final sample of 680 participants (*n* = 207 not included). Compared to the sample included, those participants not included in this nested study had lower SoC scores at W1 (*μ*_d_ = 9.66, *t*(242.16) = 5.38, *p* < 0.001), were slightly older (*μ*_d_ = 0.97 years, *t*(310.3) = 2.48, *p* = 0.014) (Welch's *t*‐tests due to significant Fisher's *F*‐tests indicating heteroscedasticity), and were more likely to be in their first pregnancy (OR_subsequent:first_ = −0.55, *Χ* ^2^(1) = 11.63, *p* < 0.001, Cramer's *V* = 0.12). However, there was no difference in frequency of depression (*Χ* ^2^(1) = 0.003, *p* = 0.96, Cramer's *V* = 0.002) between those participants who were included and those not included from the current dataset. Study protocol exclusion criteria on recruitment included lack of English language proficiency, psychiatric illness requiring acute inpatient admission, substance abuse disorder, and child protection involvement.

### 2.2 Materials and Procedure

#### 2.2.1 Sense of Coherence

To our knowledge, the Sense of Coherence Scale (also known as the Orientation to Life Questionnaire) is the only tool used to measure SoC, though it is also sometimes considered a measure of perceived meaning in life (King and Hicks 2021). It comes in the original 29‐item version, a shorter 13‐item version, 9‐item version, and brief 3‐item versions, with shortened versions all derived from the same original 29‐items (Antonovsky 1993; Ferguson et al. 2015; Schumann et al. 2003). The scale measures the degree to which an individual feels that (1) the internal and external world is structured, predictable and explicable (*comprehensible*), (2) that they possess, or have ready access to, the resources required to cope with the demands of their life (*manageable*), and (3) that such demands are perceived as manageable challenges that are worth engaging with (*meaningful*) (Antonovsky 1979, 1993).

The shorter, 13‐item version of the Sense of Coherence Scale (Antonovsky 1993) was used to assess SoC at four timepoints, in early and mid‐pregnancy and 6 and 12 months postpartum. Items are rated on a 7‐pt Likert scale with different anchors depending on the question content. For example, “Do you have the feeling that you don't really care about what goes on around you?” is rated from “Very seldom or Never (1)” to “Very often (7)” (item reflects “Meaningfulness”; reverse scored), whereas, the question “When something happened, have you generally found that:” is rated from “You overestimated or underestimated its importance (1)” to “You saw things in the right proportion (7)” (item reflects “Comprehensibility”). Scores are summed for a total SoC score at each timepoint, with a possible range of 7 to 91, and higher scores indicating better SoC. As there were no improvements in Cronbach's alpha by dropping questions for the 9‐item version of the scale (Ferguson et al. 2015), we report scores based on the 13‐item version, which is used by most studies of SoC in the perinatal period (Alcantara et al. 2023). Cronbach's alpha at each timepoint ranged from 0.87 to 0.89 in our sample.

#### 2.2.2 Depression

A diagnostic measure of depressive disorders, The Structured Clinical Interview for DSM‐IV or DSM 5 Axis I Disorders schedule (SCID) (First et al. 1997), was conducted at Wave 1. Depression was captured as a binary variable indicating a diagnosis of depression at Wave 1 or within 2 years prior to conception (=1), or no diagnosable depression within that period (=0), based on the SCID.

#### 2.2.3 Class Predictors

##### 2.2.3.1 Non‐Modifiable Demographics

Maternal age and parity were recorded at recruitment (12–20 wks pregnancy).

Childhood Trauma was retrospectively self‐reported at Wave 5 using the 28‐item brief Childhood Trauma Questionnaire (CTQ) (Bernstein et al. 2003), which results in a continuous variable indicating the frequency of abusive experiences from childhood to adolescence. Each item is scored on a 5‐pt Likert scale (1 = never true, 5 = very often true). Total scores greater than 36 indicate moderate‐to‐severe childhood trauma, whereas scores of 36 and below indicate minimal‐to‐no childhood trauma.

##### 2.2.3.2 Fulfilled Intention Variables

Four variables were created to reflect whether or not intentions during pregnancy (Wave 2) about birth and postpartum behaviors were fulfilled or not. These measures are bespoke and were constructed for this study based on our hypotheses. A score of 1 indicates that the intention at Wave 2 was fulfilled as self‐reported at Waves 4 and 5, and a score of 0 indicates that the outcome did not match the intention. These variables reflected fulfilled expectations regarding (1) pain relief during birth as a summed score to a max of 4 reflecting use of a birthing pool, transcutaneous electrical nerve stimulation (TENS), pain medication, and/or epidural; (2) mode of delivery as a binary variable (all emergency cesarean sections were coded as 0); (3) breastfeeding to a minimum duration of 12 mths as a binary variable; and (4) infant sleep location (in your bed with you, in your bed with you and your partner, in your room but not your bed, or in his/her own room) which was collapsed into intended bed sharing at 6mths as a binary variable.

##### 2.2.3.3 Postpartum Social Support

Social support at 6mths postpartum was assessed using two scales. The 12‐item Multidimensional Scale of Perceived Social Support was used to assess the perceived quality of social support received from different people, resulting in separate scores reflecting perceived support from partners, friends, and family (Zimet et al. 1990). Each item is rated on a 7‐pt Likert scale, and scores are summed for each of the three subscales (4 items each), for a total range of 4–28 per subscale. Cronbach's alphas for these subscales in our study were 0.94 (partner), 0.93 (friends), and 0.92 (family).

The quality and quantity of task support (or instrumental/practical support) and emotional support from partners, were captured as separate scores derived from a modified, 10‐item version of the Social Support Effectiveness Questionnaire (Rini and Dunkel Schetter 2010). This allows us to look not only at the “who” of social support, but also estimate the contribution of different types of social support, i.e., the “what.” Each item is scored on a 5‐pt Likert scale, scores are summed for a total possible range of 0–20 for each subscale, with increasing scores indicating more support. Cronbach's alphas for each subscale in our sample were 0.90 (task) and 0.91 (emotional).

### 2.3 Statistical Analysis

Data were aggregated and cleaned using R version 4.3.1 (R Core Team 2020). Growth mixture modeling was conducted using MPlus8 (Muthén and Muthén 2017), and other statistical analyses using IBM SPSS Statistics 29 (IBM Corp 2022).

#### 2.3.1 Group‐Level Analysis of Sense of Coherence

A mixed methods ANOVA including between‐subjects factors of depression at recruitment and parity was conducted to test for changes in SoC within subjects between waves detectable at a whole group level. If Wave had not been a significant main effect, planned pairwise comparisons of all four timepoints would have been performed, to allow for more direct comparison with previous research in SoC using fewer timepoints. If Wave was significant, pairwise posthoc comparisons would be conducted, correcting for multiple comparisons.

#### 2.3.2 Growth Mixture Modeling: Extracting Trajectory Classes

Based on previous trajectory analysis for the same larger cohort study (Porter et al. 2019), relative distances between waves were set with wave one occurring at time = 0, wave two time = 1, wave four time = 3.6, and wave five time = 5.6. This accounts for unequal temporal distance between waves. When plotted, this spacing is also used on the *x*‐axis to match the modeling.

Growth mixture modeling was selected for this study because it captures information about differences between individuals in the way their SoC changes over time, while taking a data‐driven approach to identifying groups within the broader heterogeneous population. The result is the identification of latent classes such that individuals within a class are more similar to each other in the way that their SoC changes over the measured time period than individuals in different classes, extracting a common trajectory for each of the identified classes (Jung and Wickrama 2008).

Growth models including no latent classes up to six latent classes, and those with and without a quadratic term (allowing the estimated slope of the trajectory to change over time) were systematically compared. If a model containing no latent classes were determined to be the best fitting model, then there were no discernable subgroups in SoC trajectory in the perinatal period, and if the slope and quadratic terms were also non‐significant, then this result would indicate no change in SoC in the perinatal period, consistent with previous findings from Sjöström et al. (2004) and Ngai and Ngu (2016). As such, this analysis is sensitive to detect a range of trajectories, and subgroups who follow different trajectories or lack thereof, in a data‐driven way. This is a significant advancement on previous methods used to understand SoC in the perinatal period. Depression and Parity were included in the class modeling as predictors without an interaction term, as they were identified *a priori* as possible confounders of growth trajectory of SoC in the perinatal period based on prior literature, and their influence would be seen from conception (as opposed to postpartum social support for instance, which was only included as a predictor in the subsequent regression analyses).

The best fitting model was selected based on a combination of the highest model entropy (with higher values indicating better evidence for partitioning the data in that way; Celeux and Soromenho (1996)), the lowest Akaike Information Criteria (AIC), lowest Bayesian Information Criteria (BIC), a significant Lo‐Mendell‐Rubin (LMR) test (indicating that the number of classes is better than *n *− 1), and a minimum class size of greater than 5%. Model selection using a range of criteria such as this is recommended for growth mixture modeling (Jung and Wickrama 2008).

#### 2.3.3 Predicting Trajectory Classes

##### 2.3.3.1 Univariate Analyses

To examine the independent pairwise associations between each predictor variable and the identified trajectory classes, univariate analyses were conducted. It should be noted that these analyses were done independently of the subsequent regressions, for which predictors were chosen *a priori* based on scientific hypotheses, to avoid biasing the analysis towards a significant result or missing predictors that are significantly associated with the classes only when another important variable is controlled for in the same model (Sun et al. 1996). All *a priori* selected predictor variables were included in the univariate analyses, as well as other demographic variables collected at Wave 1, specifically MPEWS Cohort, Relationship Status, University Completion, Employment, and birth outcomes, specifically Preterm birth and Neonatal Intensive Care Unit (NICU) admission. Continuous variables were analyzed using a one‐way ANOVA with class predicting each variable, and post‐hoc comparisons were Bonferroni corrected for multiple comparisons. Categorical variables were analyzed using pairwise crosstabs and a Chi‐squared test.

##### 2.3.3.2 Multinomial Logistic Regression

Four multinomial logistic regressions predicting class were constructed and compared hierarchically with each subsequent model capturing all variables in the previous model with additional predictors to assess the importance of limited unmodifiable demographic factors selected *a priori* (Maternal Age and CTQ; *Model 2*), whether the mother's intentions during pregnancy were fulfilled at birth and in the first year postpartum (regarding pain relief, mode of delivery, sleep location, and breastfeeding duration; *Model 3*), and the amount of self‐reported social support at 6mths postpartum (from partner, friends, family, and both task and emotional support; *Model 4*), relative to a base model including only the predictors included in the growth mixture modeling (Depression and Parity; *Model 1*). The order of these models and combination of variables were chosen to reflect the expected temporal sequence of their influence on trajectory of SoC in the perinatal period. The winning model was selected by comparing AIC and BIC and whether the additional variables were significant relative to *Model n − 1*. The winning model indicates the most important predictors of the trajectory of SoC in the perinatal period. To understand the influence of significant predictors, effects were compared to the largest class as reference.

## 3 Results

### 3.1 Descriptive Statistics

Mean maternal age at recruitment was 32.14 years (standard deviation (std): 4.60; range: 19–48). Most of the participants were first time mothers, in stable relationships, without depression or antidepressant use, had completed a university degree, and were working at the time of recruitment. Most of the babies born to the sample were full‐term and did not require admission to neonatal intensive care. See Table 1 for details.

**Table 1:** Participant characteristics.

Variable	Wave	Category	Frequency (valid %)
Parity	1	First Pregnancy	469 (69.0)
		Subsequent Pregnancy	211 (31.0)
Depression	1	At Wave 1 or within 2 yrs prior to conception	554 (81.5)
		No	126 (18.5)
Antidepressant use during pregnancy	1	Yes	559 (82.2)
		No	121 (17.8)
University	1	Completed Degree	433 (65.3)
		No Completed Degree	230 (34.7)
Employment status	1	Full‐time/Part‐time/Casual	562 (87.0)
		Unemployed/Parental Leave	84 (13.0)
Relationship status	1	Stable Relationship	632 (98.0)
		No Stable Relationship	13 (2.0)
Preterm birth	3	Yes	50 (7.4)
		No	626 (92.6)
Neonatal intensive care unit admission	3	Yes	54 (7.9)
		No	626 (92.1)

The majority of women in our sample had their intentions about birth and the postpartum fulfilled across the measured categories (Table 2). There are some notable groups for whom intentions during pregnancy were not fulfilled. Many women intended pain management that did not occur. Nearly one third (29%) of women who intend to breastfeed to 12 mths do not, but nearly half (47%) of women who do not intend to breastfeed to 12 mths do. The majority of women who end up bedsharing did not intend to, with 83% of women who reported bedsharing at Wave 4 not intending to bedshare when they were pregnant. Of women who intended to have a C‐section at Wave 2, 63% gave birth vaginally.

**Table 2:** Intention and outcome variable descriptive statistics.

		Not intended and did not occur	Not intended and occurred	Intended and did not occur	Intended and occurred	Intentions fulfilled frequency (valid %)
Pain Relief	Birthing Pool	617	34	13	16	633 (93.1)
	TENS	553	17	94	16	569 (83.7)
	Pain Meds^a^	324	38	295	23	347 (51.0)
	Epidural	335	37	265	43	378 (55.6)
	*Sum Total*	*Mean*: 2.83 *Standard Deviation*: 0.91 *Range*: 0–4				
Breastfeeding at 12 months		125	110	111	276	401 (59.0)
Bed Sharing at 6 months		508	62	11	12	532 (87.9)^b^

	Emergency C‐section^c^ (Unplanned)	Vaginal birth intended and occurred	Vaginal birth intended, planned C‐section occurred	C‐Section planned, vaginal birth occurred	C‐Section planned and occurred	Intentions fulfilled frequency (valid %)
Mode of Delivery	137	417	7	46	26	443 (65.1)

^a^ Pain Meds: Pain Medications administered during delivery.

^b^ Discrepancies in total for Bed Sharing capture 12 cases manually coded for intentions fulfilled based on text entry in “other” response boxes.

^c^ C‐Section: Caesarian Section.

The mean standard deviation of the Sense of Coherence measure within subjects across the perinatal period was 5.77 points (std: 3.64, range: 0–21.9). All Pearson's Correlations between SoC values across the four waves were significant, and ranged from 0.69 between Waves 2 and 5, to 0.80 between Waves 1 and 2. Mean values across participants at each wave are reported in the Supporting Materials along with descriptive statistics for the other continuous variables, and parametric and non‐parametric correlations between all variables including broader demographic variables.

After converting childhood trauma to a binary measure, 371 participants (69.5% of valid responses) reported minimal to no childhood trauma.

### 3.2 Group‐Level Analysis of Sense of Coherence

When conducting the repeated measures ANOVA, testing for changes in SoC at the group level, Mauchly's test indicated that the assumption of sphericity had been violated (*χ* ^2^(5) = 33.73, *p* < 0.001), so a Huynh‐Feldt correction was applied (*ε* = 0.964). This test showed a significant main effect of Wave (*F*(2.89, 1316.50) = 3.34, *p* = 0.02, *η*_p_^2^ = 0.007). Posthoc pairwise comparisons between waves showed a significant increase in the group mean of SoC from Wave 1 to Wave 2 (Δ*x̄* = −1.62, *p*_bonferonni_ = 0.03) and a significant decrease from Wave 2 to Wave 3 (Δ*x̄* = 1.94, *p*_bonferonni_ = 0.03). No other pairwise comparisons were significant, indicating a relative peak in SoC mid‐pregnancy at the group‐level.

For the between subjects factors, there was a significant main effect of Depression (*F*(1, 455) = 52.32, *p* < 0.001, *η*_p_^2^ = 0.10) such that participants without current or recent depression had a higher SoC than those who were depressed (Δ*x̄* = 10.67, *p*_bonferonni_ < 0.001). The main effect of Parity was insignificant (*F*(1, 455) = 3.82, *p* = 0.05, *η*_p_^2^ = 0.008).

There were no significant interactions between Depression, Parity, or Wave (0.16 < *p* < 0.42).

### 3.3 Growth Mixture Modeling: Extracting Trajectory Classes

For the both models including a quadratic term and those that didn't, there was a significant increase in model fit with when including multiple latent classes (see Table 3, significant LMR LRTs for 5‐class nonquadratic model, and 4‐class quadratic model), suggesting that there are likely subgroups present with different growth trajectories in SoC over the perinatal period. Across all tested latent class models, the largest group was a “high stable” class which captured the growth pattern for 49%–70% of participants, suggesting that while most people have a stable high SoC during the perinatal period, a sizable group do not fit this trajectory.

**Table 3:** Model comparison.

N Classes	Quadratic term?	Random slope^a^	Random Quad.^a^	Smallest class size (%)	BIC	aBIC	Entropy	LMR LRT	(*p*‐value)
1	No	Yes	N/A	100	17939.96	17898.68	N/A	N/A	N/A
2	No	Yes	N/A	29.56	17923.23	17878.78	0.66	126.36	0.06
3	No	Yes	N/A	8.38	17914.68	17854.35	0.70	39.94	0.09
4	No	No	N/A	7.94	17900.59	17830.73	0.72	41.91	0.19
5	No	No	N/A	2.35	17909.07	17823.34	0.74	23.41	**0.03**
6	No	No	N/A	2.06	17926.78	17825.17	0.72	14.49	0.24
1	Yes	Yes	Yes	100	17973.68	17913.35	N/A	N/A	N/A
2	Yes	Yes	Yes	29.56	17951.07	17890.75	0.66	127.32	0.12
3	Yes	Yes	Yes	5.74	17943.24	17863.87	0.73	45.79	0.10
4	Yes	No	No	3.38	**17899.24**	17816.69	0.77	58.74	**0.002**
5	Yes	No	No	2.35	17917.27	17815.67	**0.79**	20.58	0.44
6	Yes	No	No	2.21	17935.32	**17814.66**	0.77	20.56	0.32

^a^ When the model did not converge with a random factor for the slope or quadratic term (i.e., with those random factors the model was too complex to fit), these factors were constrained to 0.

**Figure 1:**
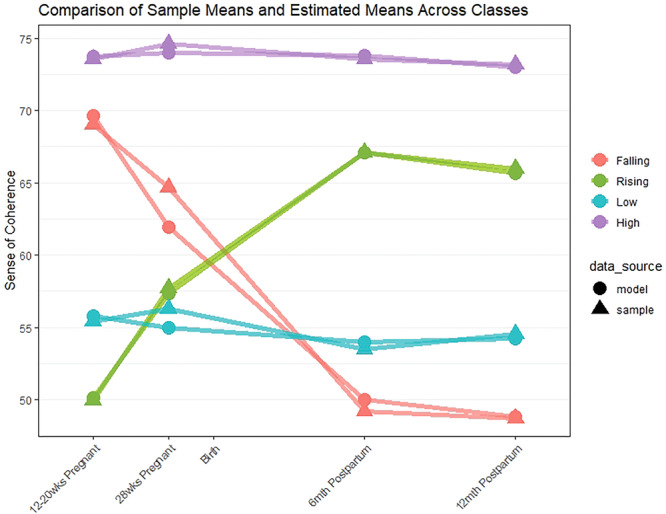
Comparison of sample means and model estimated means across classes. Falling class *n* = 23 (3.34%), Rising class *n* = 54 (7.94%), Low class *n* = 146 (21.47%), High class *n* = 457 (67.20%). Wks = weeks, mth = month.

**Figure 2:**
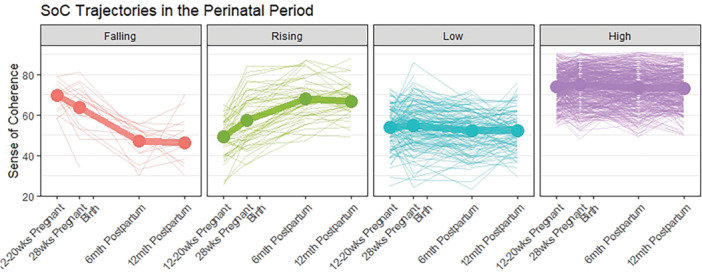
Mean trajectory (circles at each wave) and individual trajectories in each class. Falling class *n* = 23 (3.34%), Rising class *n* = 54 (7.94%), Low class *n* = 146 (21.47%), High class *n* = 457 (67.20%). Wks = weeks, mth = month (*Note:* not all classified individuals are plotted, participants were omitted when available datapoints did not allow any connections between adjacent timepoints).

Based on the smallest BIC, the second highest entropy, and a significant LMR LRT test statistic, the winning model has four classes and includes a quadratic term (Table 3; Figure 1). While the smallest class is less than 5% of the total sample, it includes 23 participants, which is considered of sufficient size for generalization to a broader sample in the future (Table 4), and the four‐class model is overall preferable to the three‐class model despite this limitation (significant LMR LRT). The four classes in this model, in order of decreasing size, can be labeled “High,” “Low,” “Rising,” and “Falling” (Figure 2). The High and Low classes have significant intercepts, but no significant slope or quadratic term (Table 4), indicating that the trajectory of SoC in these groups is relatively flat across the perinatal period. In contrast, both the slope and quadratic terms in the Falling and Rising classes are significant (Table 4), indicating both a change in mean SoC across the four timepoints, and a change in the size of this change over the perinatal period. In both cases, the change in SoC decreases over time, to a fairly flat trajectory between Waves 4 and 5 (6–12 mths postpartum). This indicates that when SoC fluctuates in the perinatal period (which happens for 11.28% of individuals), the greatest changes in SoC occur during pregnancy and before 6mths postpartum. The means for each timepoint in each of the four classes as compared to their estimated model means can be seen in Figure 1, and the individuals in each class as compared to the class means is depicted in Figure 2. The model underestimates the SoC in Wave 2 for the Falling and Low classes in particular, but otherwise appears to capture the trajectories well.

**Table 4:** Winning latent class model: Four classes with depression and parity binary predictors and quadratic term.

Class	Label	Size *N* (%)	Intercept (Wave 1) mean (95% CI)	Slope mean (95% CI)	Quadratic mean (95% CI)
1	Falling	23 (3.34)	69.66 (66.82:72.50)**	−8.57 (−12.02:−5.12)**	0.87 (0.20:1.53)*
2	Rising	54 (7.94)	50.13 (46.35:53.91)**	8.20 (6.44:9.96)**	−0.97 (−1.30:−0.64)**
3	Low	146 (21.47)	55.75 (52.97:58.54)**	−0.87 (−2.15:0.42)n.s	0.11 (−0.13:0.34)n.s
4	High	457 (67.20)	73.76 (72.59:74.93)**	0.27 (−0.24:0.77)n.s	−0.72 (−0.16:0.02)n.s

* *p* < 0.05

** *p* < 0.001.

^n.s^ *p* > 0.05.

### 3.4 Predicting Trajectory Classes

#### 3.4.1 Univariate Analyses

Chi‐Square tests showed significant associations between SoC trajectory in the perinatal period and Depression at recruitment or within the 2 years prior to conception, Parity, Relationship Status, University completion, and Employment (see Table 5). Inspecting the cross‐tabulation distributions, more depressed participants fell in the Low (67.5% vs. 11%) or Rising (14.3% vs. 6.5%) classes than the non‐depressed participants. Multiparity appears to increase the likelihood of falling in the Low class (28% vs. 18.6%), and decreased the likelihood of being in the Rising class (5.2% vs. 9.2%). Not being in a stable relationship at recruitment was more common in the Low class (23.1% vs. 2.8%) and the Falling class (30.8% vs. 20.9%), whereas those in a stable relationship were more often in the High class (68.2% vs. 38.5%). Completion of a university degree was more often seen in the High class (71.4% vs. 58.3%), with increased proportions in all other classes for those without. Working full‐time, part‐time or casually at recruitment was also associated with the High class (69.2% vs. 51.2%) with no paid employment increasing the likelihood of being in all other classes.

**Table 5:** Chi square tests of univariate association between class and categorical predictors.

Predictor	*N*	*df*	Pearson *X* ^*2*^	*p*‐value
Depression	680	3	220.44	**< 0.001**
Parity	680	3	9.50	**0.02**
Cohort	680	9	10.23	0.33
Relationship Status	645	3	18.27	**< 0.001**
University	663	3	11.85	**0.008**
Employment	646	3	11.93	**0.008**
Preterm Birth	676	3	2.09	0.56
NICU	680	3	1.18	0.76
MOD IF	633	3	1.72	0.63
Breastfeeding IF	622	3	4.41	0.22
Bed Sharing IF	605	3	6.64	0.08
Sleep Location IF	605	3	0.80	0.85

*Note:* Bold indicates significant results with *p* < 0.05.

Abbreviations: IF, intention fulfilled; MOD, mode of delivery; NICU, neonatal intensive care unit.

The one‐way ANOVA results show that all forms of postpartum social support have a moderate effect on SoC trajectory class membership (see Table 6). Greater Partner support is associated with being in the High class as opposed to the Low class (∆*x̄* = 0.53, *p* < 0.001). Increased Family (∆*x̄* = 0.90, *p* < 0.001) and Friend (∆*x̄* = 0.82, *p* < 0.001) support was also observed in the High class relative to the Low class, but also being in the Rising class as opposed to the Low class (Family: ∆*x̄* = 0.69, *p* = 0.009; Friend: ∆*x̄* = 0.59, *p* = 0.02). In terms of domain of support, increased Task support was associated with being in the High class as opposed to the Falling (∆*x̄* = 3.32, *p* = 0.002) or Low (∆*x̄* = 2.70, *p* < 0.001) classes. Greater emotional support was seen in the High class than the Falling class (∆*x̄* = 4.06, *p* < 0.001) and the Low class (∆*x̄* = 2.88, *p* < 0.001), as well as in the Rising class as compared to the Falling (∆*x̄* = 3.13, *p* = 0.03) or Low classes (∆*x̄* = 1.96, *p* = 0.04). In addition, greater maternal childhood trauma, as measured by CTQ, is associated with the Low (∆*x̄* = 8.27, *p* < 0.001) and Falling (∆*x̄* = 11.06, *p* = 0.001) classes as compared to the High class.

**Table 6:** One‐way ANOVA results of univariate association between class and continuous variables.

Variable	*df*	*F*‐value	*p* value	Eta‐squared
Maternal Age	(3, 676)	2.18	0.09	0.03
CTQ	(3, 531)	17.14	**< 0.001**	0.13
Pain Relief IF	(3, 676)	2.54	0.06	0.03
Social Support—Partner	(3, 637)	11.11	**< 0.001**	0.08
Social Support—Family	(3, 637)	16.26	**< 0.001**	0.11
Social Support—Friends	(3, 637)	17.20	**< 0.001**	0.11
Social Support—Task	(3, 629)	17.11	**< 0.001**	0.11
Social Support—Emotion	(3, 613)	18.66	**< 0.001**	0.12

*Note:* Bold indicates significant results with *p* < 0.05.

Abbrevitaion: IF, intention fulfilled.

#### 3.4.2 Multinomial Logistic Regression

##### 3.4.2.1 Model Selection

Since there were no participants in the Falling class that did not have their binary bed‐sharing intentions fulfilled, the original and more complex four‐location sleeping intention‐outcome variable was used for Models 3 and 4 to avoid infinite parameter estimates.

The preferred model is Model 3, including the base model factors of Depression and Parity, as well as demographic factors (Maternal Age and CTQ) and the intention‐outcome variables. This model had the lowest BIC, and is not improved by adding social support variables (none of which significantly contributed to the larger Model 4). Results for the other models are reported in the Supporting Materials.

##### 3.4.2.2 Winning Model: Depression, Parity, Age, CTQ, Fulfilled Intention Variables

The model including binary fulfilled intention variables showed lower AIC and BIC values than Model 2 (Depression, Parity, Age and CTQ only), indicating better fit (AIC = 653.03, BIC = 763.68), and accounts for approximately 35% percent of variance in the data (Nagelkerke *R* ^2^_pseudo_ = 0.35). Parameter estimates showed that CTQ (*X* ^*2*^(3) = 24.48, *p* < 0.001) and Depression (*X* ^*2*^(3) = 95.35, *p* < 0.001) significantly contributed to the model, with Depression increasing the likelihood of being in the Rising (*B* = 2.16, *p* < 0.001) or Low (*B* = 3.30, *p* < 0.001) classes, and increased childhood trauma increasing the likelihood of being in the Falling (*B* = 0.05, *p* = 0.008) or Low (*B* = 0.05, *p* < 0.001) classes relative to the High class. Despite increasing the overall accuracy of the model relative to both simpler and more complex models, none of the intention‐outcome variables significantly contributed to the model's predictions of SoC trajectory class (with *p*‐values ranging from 0.053 for pain relief intentions to 0.84).

## 4 Discussion

In this study we aimed to determine whether SoC changes across four timepoints in the pregnancy and the postpartum, whether there are distinct patterns of SoC trajectory in the perinatal period and if there are, what predicts which trajectory an individual will follow. At the whole group level, our results suggest a relative peak in SoC at 28 weeks gestation, as compared to early pregnancy (12–20 wks) and 6 mths postpartum. This is largely inconsistent with previous findings that SoC increases between pregnancy and the early postpartum (Bäckström et al. 2018; Ferguson et al. 2016; Hildingsson 2017), but is consistent with findings of an increase in sense of self (including agency and self‐efficacy) in the second trimester of pregnancy (Perrykkad et al. 2024). The growth mixture modeling analysis, which aimed to identify subgroups and classify individual trajectories, suggests that while most people have a fairly stable SoC even in transformational periods like the perinatal period, 1 in 10 people have fairly drastic changes to SoC during this time, which mostly restabilises at the new value between 6 and 12 mths postpartum. We determined that four classes were the best fit to the data, with a High and stable (67%), Low and stable (21%), Rising (8%), and Falling (3%) classes. In both of the changing classes, the biggest changes occur between early pregnancy and 6mths postpartum, with all classes exhibiting a fairly flat trajectory 6–12 mths postpartum, further evidenced by the significance of a quadratic term in these classes which improved model fit.

In predicting whether a person's trajectory in Sense of Coherence across pregnancy and into the first year postpartum will be High, Low, Rising, or Falling, with class definitions taking into account whether or not people meet the criteria for depression in early pregnancy or within 2 years postpartum and whether the mother is multiparous, the most important factors are recent pre‐conception or early pregnancy depression and a history of childhood trauma. Mothers with more childhood trauma were more likely to have a Falling or Low SoC in the perinatal period than High SoC. While depression in early pregnancy or the 2 years preceding conception was taken into account in the data‐driven definition of the trajectory classes, it did not significantly contribute to the prediction of all of the classes. Depression was associated with an increased likelihood of having a Rising or Low trajectory in SoC in the perinatal period. Combined, these findings suggest that increased childhood trauma, in the form of physical, sexual, and emotional abuse, and physical and emotional neglect, is more predictive of a poor sense of coherence at the end of the first year postpartum. This suggests that the perinatal period has a negative impact on their generalized resistance resources (i.e., coping strategy arsenal allowing for rational, flexible, and farsighted response to managing or avoiding stressors (Antonovsky 1979)), whereas depression in early pregnancy is more predictive of the starting point of sense of coherence than its trajectory over the perinatal period.

Previous research suggests that individuals with a history of childhood trauma have a lower SoC than those without (Renck and Rahm 2005; Watts 2022) including into later life (Naderzadeh et al. 2023; van der Hal‐van Raalte et al. 2008), and during pregnancy (Finnbogadóttir et al. 2020). To our knowledge, this is the first study to show an effect of childhood trauma on the trajectory of SoC through a transformative experience, and our results suggest that in addition to leading to an overall low SoC, childhood trauma may predispose people to declining SoC in the face of changing circumstances. Childhood trauma may be internalized as an expectation of unpredictability and inconsistency, in treatment by adults (as in the case of sexual, emotional and physical abuse), or in available resources to meet the psychological and physical needs of the child (as in cases of physical and emotional neglect). While these may be thought to affect the setpoint of SoC (and thus the starting point of SoC in the perinatal period), our findings are more consistent with previous research that suggests that childhood trauma may sensitize an individual's reactions to future instability by weakening SoC (Fossion et al. 2014). This is consistent with our hypothesis that childhood trauma may affect the comprehensibility component of SoC in the perinatal period, as many new experiences of pregnancy, birth and parenthood occur and must be psychologically processed.

While usually being considered a stable, lifelong trait, SoC is thought to be more flexible during times of “radical change in one's structural situation” (Antonovsky 1979, p. 125). Given that the predictability of one's life circumstances constitute the comprehensibility subscale, it was hypothesized that pregnant individuals whose expectations of birth and the postpartum were not met may be more likely to show a decline in SoC over the perinatal period. This was not borne out in the results, with none of the fulfilled intention variables significantly contributing to predicting SoC trajectory. Similarly, it was thought that complications during birth may reduce SoC, but our univariate analyses did not show a significant relationship. While this may be because the majority of infants in our sample were full‐term, our findings are aligned with findings from Larsson et al. (2009), who found no difference in SoC before and after abnormal ultrasound scans. This may be because SoC itself provides a buffer to stress and negative emotions following such news, and may not be sensitive to events with a short duration, even if negative in nature. Conversely, Habroe et al. (2007) found that achieving childbirth after using assisted reproductive technologies was associated with an increase in SoC, suggesting that achieving a long sought after life goal may increase SoC itself. More research is needed to understand how significant events (including but not limited to reproductive events) in the perinatal period interact with SoC and its trajectory.

Despite finding no relationship between antenatal expectations for delivery and early parenting being fulfilled and SoC trajectory, it is reassuring to see that across all measured categories (pain relief at birth, mode of delivery, breastfeeding to 12 mths, and sleep location), women tend to have the outcomes they planned in the perinatal period, and when this does not occur, the outcomes were not poorer and included longer breastfeeding duration than intended and a normal vaginal delivery. While there is no previous research that we know of investigating the relationship between unfulfilled expectations of the perinatal period and SoC, there is some research to show links with perinatal depression (Kahalon et al. 2022), relationship adjustment (Harwood et al. 2007), and quality of life (Adams et al. 2021). As such, there may be interesting avenues for future research to consider the different kinds of expectations and more complex relationships with such other factors or subscales of the SoC such as comprehensibility.

Possible limitations of this study include the temporal overlap in the assessment of the predictor variables and SoC, which were part of the same batteries of questionnaires (e.g., depression at wave 1, childhood trauma retrospectively assessed at wave 5, at which times SoC data was also collected). However, none of the predictors was assessed longitudinally (while the fulfilled intention variables involved elements across time, they are each only assessed once), so this is unlikely to confound relationships with SoC trajectory. Future research may consider temporally separate assessments to ensure complete independence from, e.g., covariance due to participant mood during survey completion.

Another limitation is the small size of the falling class (<5%, *n* = 23). In setting our criteria for a winning growth mixture model we aimed to have no class size smaller than 5% of the total sample but our winning model did not achieve this and was selected on a constellation of other factors, in line with Jung and Wickrama (2008). The concern is that having a class size so small limits the generalizability of the model to other samples and the broader population, and may result in false negative or false positive findings with respect to the subsequent multinomial logistic regression analyses as spurious findings are more likely in smaller samples. Conceptually, individuals with a falling trajectory are the ones that are most vulnerable and have the greatest potential to benefit from clinical intervention, since we know they *can* have a high SoC, and interventions may be able to prevent or slow its fall. Given their vulnerability, it would be reassuring to find that few individuals are at such a risk. While targeting individuals with stable and low SoC would also be admirable, their setpoint may be more difficult to shift. Regardless, further research into the generalizability of our findings to other groups is important to establish the feasibility of developing targeted interventions to help individuals with falling trajectories particularly.

The consideration of assessing SoC in the perinatal period (during pregnancy and early in the postpartum) requires replicated and expanded research to determine if including this would make a meaningful contribution to the current comprehensive screening and assessment of women within antenatal care. Based on our results, we support the current recommended antenatal mental healthcare screening (e.g., for depression) and targeted assessment of maternal history of trauma as critical during pregnancy. While direct assessment of SoC is not at this stage recommended in clinical care settings, these results suggest that individuals with a known history of depression at the beginning of pregnancy may particularly benefit from timely treatment that may also include a focus on building a sense that the world is manageable, meaningful, and comprehensible (Muggleton et al. 2021). The benefits of a high SoC include increased quality of life (Eriksson and Lindström 2007), better mental and physical health (Eriksson and Lindström 2006; Matić and Vuletić 2023), increased health seeking behavior (Binkowska‐Bury et al. 2016), and more resilient responses to personal challenges (Richardson and Ratner 2005; van der Hal‐van Raalte et al. 2008). Further, our results support previous findings that women who have a history of childhood trauma are particularly vulnerable during this period, and as such the importance of trauma‐informed maternity care. While currently there are no evidence‐informed interventions for SoC, embedding salutogenic principles in maternity services such as improving predictability, informed‐consent, choice, and meaning, where possible, will support SoC in all women.

## 5 Conclusion

The finding that most (but not all) people have stable SoC in the perinatal period is consistent with previous studies (Alcantara et al. 2023), but highlights the importance of considering factors which may lead to more fluctuation. By identifying that the primary factors that influence SoC trajectory in the perinatal period are maternal childhood trauma and depression, our study redoubles the importance of mental health support in the perinatal period to ensure individuals have adequate coping resources to weather the transformative storm.

## Consent

The study was approved by the Human Research Ethics Committees of Mercy Health (R08/22), the South Metropolitan Health Service (REG Number: 2016‐192), and the Western Australia Country Health Service (18/02) and was conducted in accordance with the approved protocols and the Australian National Health and Medical Research Council's National Statement on Ethical Conduct in Human Research. All participants provided written informed consent.

## Conflicts of Interest

The authors declare no conflicts of interest.

## Supporting information

Supporting File 1

Supporting File 2

## Data Availability

The study's original data is confidential and is only available to investigators on the study under the conditions of the ethics approval. However, the study does have a protocol for proposals to use data within the study in collaboration with Investigators and proposals can be addressed to Professor Megan Galbally megan.galbally@monash.edu and will then be reviewed by the key investigator team. This study was not preregistered.
