# Supplementary material for: Sense of Coherence in the Perinatal Period: A Longitudinal Growth Mixture Modeling Analysis
Source: J Clin Psychol. 2026 Jun 6;82(9):1271–84. doi: 10.1002/jclp.70156 (PMC13441375; doi:10.1002/jclp.70156)
Supplement: Supplementary file 1 [file JCLP-82-1271-s001.docx]

# Supplementary Material:

## For the correlation matrix between variables, see Correlations.xlsx also attached.

## Continuous Variables Descriptive Statistics

| **Variable** | **Wave** | **Mean** | **Standard Deviation** | **Range** |
| --- | --- | --- | --- | --- |
| Sense of Coherence | 1 | 67.19 | 12.81 | 25-91 |
|  | 2 | 68.76 | 13.04 | 24-91 |
|  | 4 | 67.56 | 13.28 | 23-91 |
|  | 5 | 67.89 | 12.75 | 29-91 |
| Childhood Trauma | 5 | 34.81 | 12.15 | 1-116 |
| Social Support – Partner | 4 | 6.48 | 1.07 | 1-7 |
| Social Support – Family | 4 | 5.71 | 1.49 | 1-7 |
| Social Support – Friends | 4 | 5.79 | 1.32 | 1-7 |
| Social Support – Task | 4 | 12.70 | 4.33 | 0-20 |
| Social Support – Emotional | 4 | 13.76 | 4.50 | 0-20 |

## Multinomial Logistic Regression Non-winning Model Results

#### Model 1 (Base Model): Depression and Parity

The base model including only the variables used as predictors in the growth mixture modelling showed a good fit to the data (Pearson *X^2^* (3, *N* = 680) = 2.03, *p* =.57; AIC = 65.87, BIC = 276.07), and accounts for approximately 32% of variance in the data (Nagelkerke R^2^_pseudo_ = .32). Depression contributed significantly to the model (*X^2^*(3) = 199.18, p < .001), with depression increasing the likelihood of being in the Rising class (B = 2.34, p < .001) or the Low class (B = 3.36, p < .001) as opposed to the High class. Parity was marginally significant (*X^2^*(3) = 7.91, p = .048), with multiparity increasing the likelihood of being in the Low class relative to the High class (B = .48, p = .045).

#### Model 2: Depression, Parity, Age, CTQ

The model including demographic factors showed a poor fit to the data (Pearson *X^2^* (1335, *N* = 535) = 1442.47, *p* = 0.021; AIC = 750.20, BIC = 814.43), however, for Models 2-4, SPSS also reported a high number of cells with zero frequencies across combinations of predictors, which invalidates the model fit tests. The model accounts for approximately 33% of variance in the data (Nagelkerke R^2^_pseudo_ = .33). As in the first model, Depression (*X^2^*(3) = 116.56, p < .001) significantly increased the likelihood of being in the Rising (B = 2.04, p < .001) or Low classes (B = 3.09, p < .001). CTQ score also significantly contributed to the model performance (*X^2^*(3) = 31.87, p < .001), with higher scores increasing the likelihood of being in the Falling class (B = 0.06, p < 0.001) and the Low class (B = 0.05, p < 0.001) relative to the High class. Neither Parity (p = 0.08) nor Maternal Age (p = 0.36) significantly contributed to the model.

#### Model 4: Depression Parity, Age, CTQ, Intention-Outcome Variables, Postpartum Social Support

The model including social support showed lower AIC but higher BIC values than Model 3 (AIC = 638.77, BIC = 809.06), and accounts for approximately 39% percent of variance in the data (Nagelkerke R^2^_pseudo_ = 0.39). Depression (*X^2^*(3) = 71.91, p < .001), CTQ (*X^2^*(3) = 8.30, p = .04) and number of pain intentions fulfilled (*X^2^*(3) = 7.84, p = .050) contributed significantly to model predictions in the same way as Model 3, but none of the postpartum social support variables significantly added to the model quality (.34 < p < .84).
